# Correlations between 3D preoperative planning and postoperative reduction in the osteosynthesis of distal humeral fractures

**DOI:** 10.1186/s13018-023-03772-y

**Published:** 2023-04-08

**Authors:** Yuichi Yoshii, Sho Iwabuchi, Akira Ikumi, Sho Kohyama, Takeshi Ogawa, Tomoo Ishii

**Affiliations:** 1 Department of Orthopaedic Surgery, Tokyo Medical University Ibaraki Medical Center, 3-20-1 Chuo, Ami, Inashiki, Ibaraki 300-0395 Japan; 2 Department of Orthopaedic Surgery, University of Tsukuba Hospital, Tsukuba, Ibaraki 305-8576 Japan; 3 Department of Orthopaedic Surgery, Kikkoman General Hospital, Noda, Chiba 278-0005 Japan; 4 Department of Orthopaedic Surgery, Mito Medical Center Hospital, Ibaraki, Ibaraki 311-3193 Japan

**Keywords:** Distal humerus fracture, Preoperative plan, Computed tomography, Three dimensions, Osteosynthesis, Computer-assisted orthopedic surgery

## Abstract

**Background:**

Three-dimensional preoperative planning has been applied to the osteosynthesis of distal humerus fractures. The present study investigated the correlations between 3D preoperative planning and postoperative reduction for the osteosynthesis of distal humerus fractures using 3D parameters.

**Methods:**

Twenty-three elbows of 23 distal humerus fracture patients who underwent osteosynthesis with three-dimensional preoperative planning were evaluated. 3D images of the distal humerus were created after taking preoperative CT scans of the injured elbow. Fracture reduction, implant selection, and placement simulations were performed based on 3D images. Postoperative CT images were taken 1 month after surgery. Correlations were evaluated with preoperative plans and postoperative 3D images. The longitudinal axis and coordinates of the humerus were defined on the 3D images. The coronal angle (CA) was defined as the angle formed by the long axis and the line connecting the medial and lateral margins of the trochlea of the humerus on a coronal plane image. The sagittal angle (SA) was defined as the angle formed by the long axis and the line connecting the top of the lateral epicondyle and the center of the humeral capitellum on a sagittal plane image. The axial angle (AA) was defined as the angle between the sagittal plane and the line connecting the medial and lateral margins behind the trochlea of the humerus. The intraclass correlation coefficients (ICC) of each measurement value were assessed between preoperative planning and postoperative images.

**Results:**

Preoperative planning and postoperative measurement values were CA: 85.6 ± 5.9°/85.8 ± 5.9°, SA: 140.9 ± 8.5°/139.4 ± 7.9°, and AA: 84.0 ± 3.1°/82.6 ± 4.9°, respectively. ICCs were CA: 0.75 (*P* < 0.01), SA: 0.78 (*P* < 0.01), and AA: 0.34 (*P* < 0.05), respectively.

**Conclusions:**

The 3D preoperative planning of distal humeral fractures achieved the good correlations of coronal and sagittal angles, but the relatively poor correlation of the axial angle. This may be attributed to an inability to assess the rotation angle during surgery. We propose the measurement indices shown in the present study as a three-dimensional evaluation index for distal humerus fractures.

***Trial registration*:**

Registered as NCT04349319 at ClinicalTrials.gov.

## Background

A distal humerus fracture is a fracture of the distal end of the humerus, one of the three bones (the humerus, radius, and ulna) that make up the elbow joint. Fractures of the distal humerus in adults account for 2% of all fractures and approximately 30% of all humeral fractures [1–4]. Anatomically, the distal humerus has a triangular shape that comprises two columns and a tie arch [4, 5]. The medial column holds at its distal end of the non-articular medial epicondyle with the insertion of the flexor muscles and the medial part of the humeral trochlea. The lateral column holds at its distal end of the capitellum and more proximally at the lateral epicondyle with the insertion of the extensor muscles.

Open reduction and internal fixation has become the standard treatment for distal humerus fractures [6–9]. The aim of surgical treatment is to reconstruct the strong triangular structure at the distal humerus [10]. Rigid internal fixation and anatomical remodeling are essential for the recovery of elbow function, bone healing, and the avoidance of cartilage degeneration [11]. Regarding rigid fixation, biomechanical studies demonstrated the advantages of double plating over single plating in metaphysis and intraarticular fractures of the distal humerus [12–15]. However, the number of screws that may be inserted into distal humerus fragments is limited due to the interference of screws inserted from the medial and lateral plates.

The utility of a three-dimensional (3D) surgical simulation has recently been reported [16–19]. Evaluations of 3D bone morphology and preoperative planning are considered to be an effective means for increasing the accuracy of surgery and reducing complications. In a previous study, a 3D preoperative planning system was developed to manage fractures around the elbow [20]. This system allows the reduction process and implant placement/choices to be visualized in a virtual space. It has the advantage of being able to predict in advance the interference of screws inserted from the medial and lateral plates. However, a method has not yet been established to three-dimensionally evaluate the reduction shape accuracy of the 3D preoperative plan. In the present study, we developed a method to evaluate reduction shape accuracy based on the 3D coordinates of the distal humerus. Using this method, the correlations between 3D preoperative planning and postoperative reduction were evaluated in the osteosynthesis of distal humerus fractures. We hypothesized that 3D preoperative planning for osteosynthesis of distal humeral fractures would have good correlations between 3D preoperative planning and postoperative reduction with an assessment of 3D parameters.

## Methods

This study protocol was approved by the Institutional Review Board (approved No. 14-21, T2022-0041). The present study was registered as NCT04349319 at ClinicalTrials.gov. This was a prospective case series (level of evidence II). Twenty-three elbows of 23 distal humerus fracture patients who underwent osteosynthesis with 3D preoperative planning (14 females, 9 males, mean age 61.3 years, age range 21–87) were evaluated. Written consent was obtained from all study participants. Patients were excluded if they had a previous history of traumatic arm injuries. All patients had CT images of the injured elbow taken before and 1 month after surgery. According to preoperative X-ray (posterior-anterior and lateral view) and CT scans, fractures were classified using the AO classification system. CT images were taken with tube settings of 120 kV and 100 mAS, a section thickness of 0.8 mm, and a pixel size of 0.3 × 0.3 mm (Sensation Cardiac, Siemens). Images were taken in a range of approximately 20 cm centered on the elbow joint.

### 3D preoperative planning

**Fig. 1:**
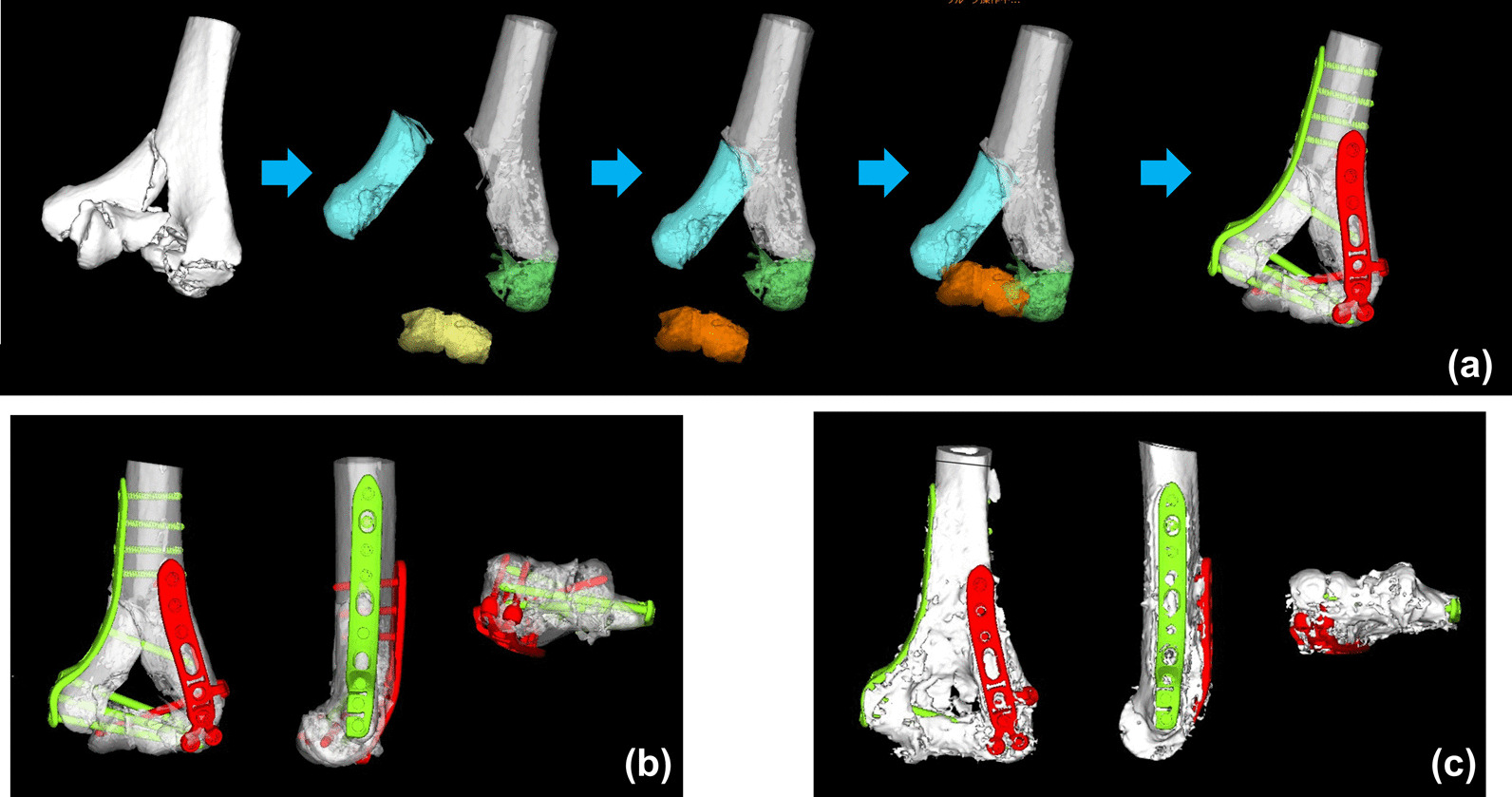
An example of the preoperative planning process. **a** Reduction and implant placement simulation. **b** Completed preoperative plan. **c** Postoperative images

3D preoperative planning and a surgical simulation were performed prior to surgery (Fig. 1). Preoperative planning software (Zed-Trauma Distal Humerus Stage, LEXI Co., Ltd., Tokyo, Japan) was used for the reduction and implant placement simulation. A 3D image of the distal humerus was created from the DICOM data of CT scans. A fracture reduction simulation was performed by separating bone fragments along the fracture line. Each distal humerus fragment was segmented according to the fracture line. The main reduction criteria were the improvement of shortening, angular and rotational deformation, the recovery of joint surface compatibility, and connecting between bone fragments. After repositioning the fragment, we confirmed the 3D shape of the distal humerus. Fragments larger than 10 mm were considered for reduction, while smaller ones were excluded from the reduction simulation. After reduction, simulations of implantation with the locking plates and screws of various sizes were performed. Computer-aided design models of different-sized implants were installed in the software. Criteria for plate selection were as follows: (1) the proximal portion of the plate reaches the diaphysis beyond the fracture line, allowing at least 3 screws to be inserted into the diaphysis; (2) enables screw fixation to the major distal fragments; (3) the insertion direction or length of the distal screw does not perforate the articular surface. Distal screws that were long enough to support distal humeral fragments were selected. Regarding proximal screws, screws of sufficient lengths were selected to reach the contralateral bone cortex. After reduction and implantation simulations, osteosynthesis was performed under general anesthesia. Surgery was conducted in the lateral position with the injured side up, and the injured limb was fixed by placing a support table under the elbow. A posterior approach to the elbow joint was used to expand the fracture site. Osteotomy of the olecranon was added where necessary to reconstruct the articular surface. During surgery, the surgeon performed reduction and the placement of implants while comparing images between the preoperative plan and fluoroscopy images obtained during surgery. The positions of the plates were selected based on the distance from the articular surface of the distal end of the plate and fluoroscopic images. Screw lengths were selected by intraoperative depth gauge measurements with reference to preoperative measurements. Surgeries were performed by nine trainees (residents and fellows) and one hand surgeon. The hand surgeon participated in all surgeries.

### Evaluations

**Fig. 2:**
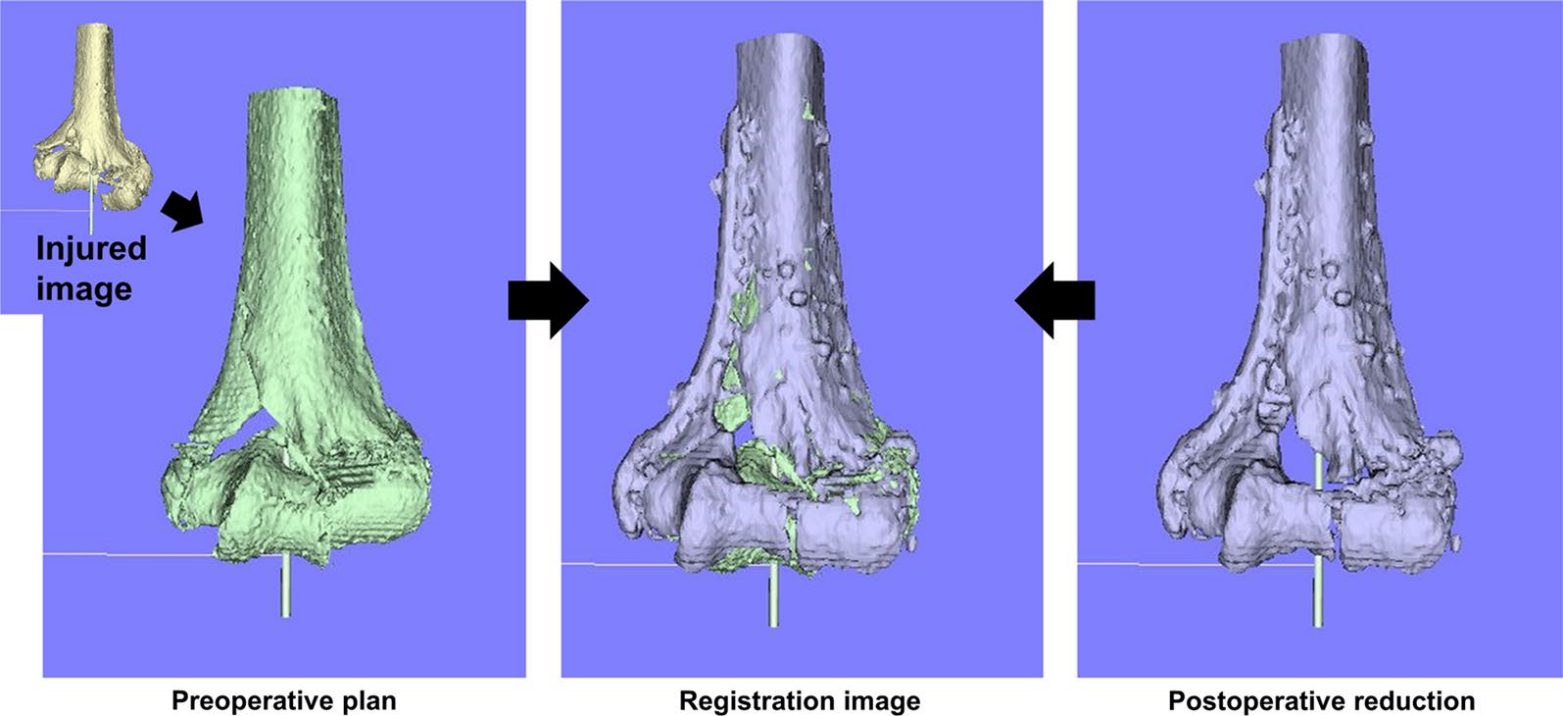
An example of a registration image for the coronal view

Preoperative and postoperative 3D images of the distal humerus were analyzed with image analysis software (BoneSimulator, Orthree, Osaka, Japan). After importing image data into the software, a surface construction algorithm was used to construct a 3D surface model of the humerus (Fig. 2). The long axis of the humerus was calculated from a preoperative 3D surface model of the intact portion of the humerus. An intact portion of the distal humerus image was used for registration between the preoperative planning image and postoperative reduction image. The coronal plane is parallel to the long axis of the humeral shaft and includes the long axis and passes through the top of the medial epicondyle, the sagittal plane is the plane including the long axis and perpendicular to the coronal plane, and the plane perpendicular to the long axis is the axis defined as a cross-section. The origin of coordinates was defined as the intersection of the joint surface and the humerus long axis on the preoperative plan image. Preoperative planning and postoperative 3D models were evaluated in the same coordinate system.

**Fig. 3:**
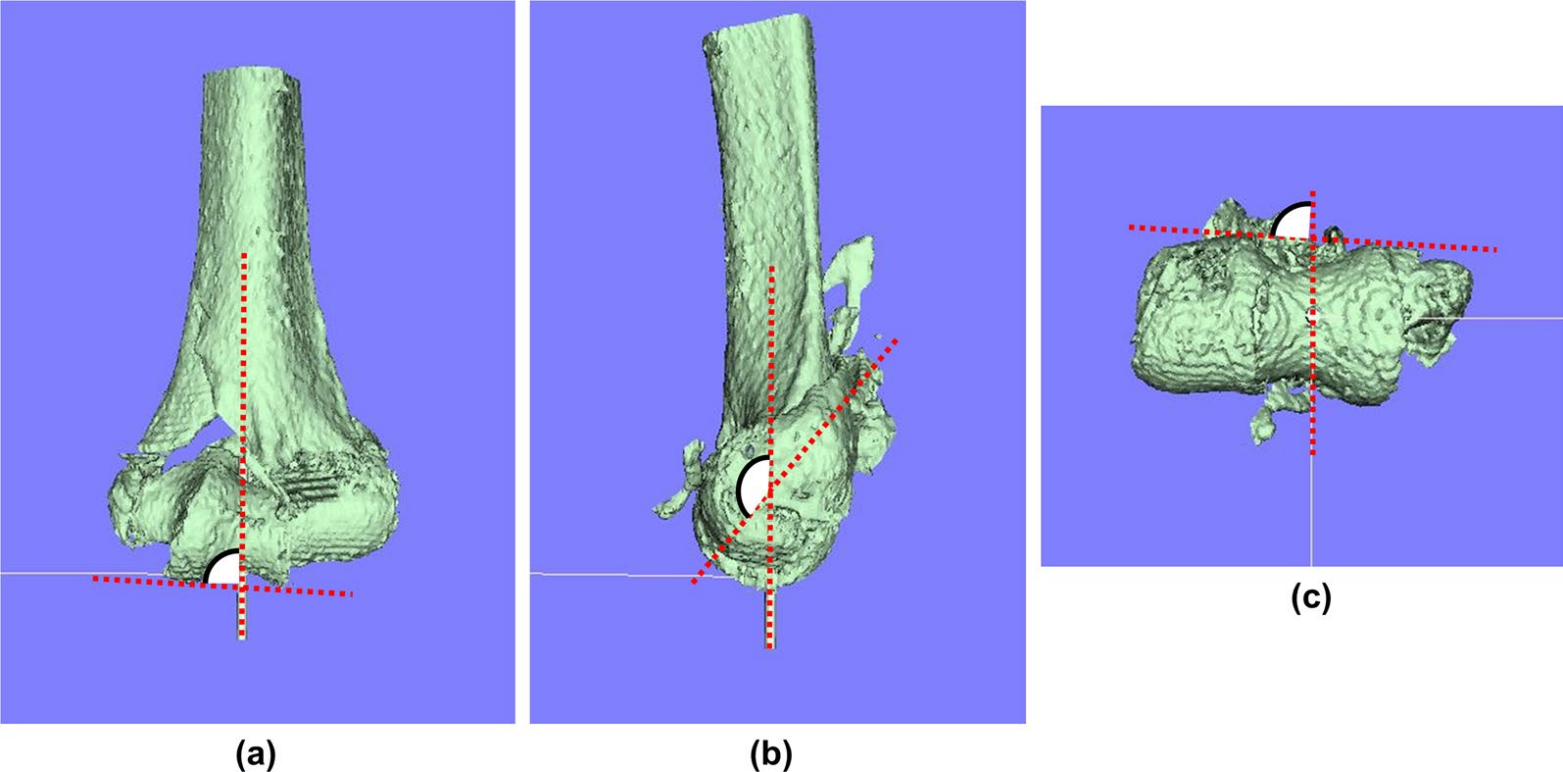
3D parameters. **a** Coronal angle (CA). **b** Sagittal angle (SA). **c** Axial angle (AA)

In the correlation analysis, three angular parameters were measured according to anatomical landmarks (Fig. 3). The coronal angle (CA) was defined as the angle formed by the long axis and the line connecting the medial and lateral margins of the trochlea of the humerus on the coronal plane image. The sagittal angle (SA) was defined as the angle formed by the long axis and the line connecting the top of the lateral epicondyle and the center of the humeral capitellum on the sagittal plane image. The axial angle (AA) was defined as the angle between the sagittal plane and the line connecting the medial and lateral margins behind the trochlea of the humerus. Each parameter was measured on preoperative planning and postoperative images. Two raters independently assessed images. One rater was involved in the surgeries and the other was not involved in the surgeries. After evaluating the reliability of the two raters’ measurements, the mean values for each parameter were used in further analyses.

### Statistical analysis

In this study, we assessed the sample size with non-parametric binominal reliability demonstration test. For the calculation, we set the number of allowable test failures: 1, reliability: 80%, test confidence level: 95%. Subsequently, the sample size was determined as 22. Results are expressed as the mean ± standard deviation. The Shapiro–Wilk test was used to test the normality of datasets. Interrater reliability was assessed using the intraclass correlation coefficient (ICC). In addition, the ICCs for the parameters between the preoperative plan and postoperative reduction were assessed. According to the previous recommendation, ICC values less than 0.5, between 0.5 and 0.75, between 0.75 and 0.9, and greater than 0.90 were defined as poor, moderate, good, and excellent correlations, respectively [21]. *P* values < 0.05 were considered to be significant. All analyses were performed using BellCurve for Excel version 2.12 (SSRI Co., Tokyo, Japan).

## Results

There were six patients with A2 fractures, four with A3 fractures, six with C2 fractures, and five with C3 fractures. There was one case each of B1 and B2 fractures. Screw fixation was performed in two cases. Single plate fixation was conducted in five cases (lateral plate: two cases, medial plate: two cases, postero-lateral plate: one case). Double plate fixation was performed in 16 cases (combination of medial and lateral plates: 12 cases, combination of medial and postero-lateral plates: four cases). The mean surgical time was 203.2 min (105–335 min).

**Fig. 4:**
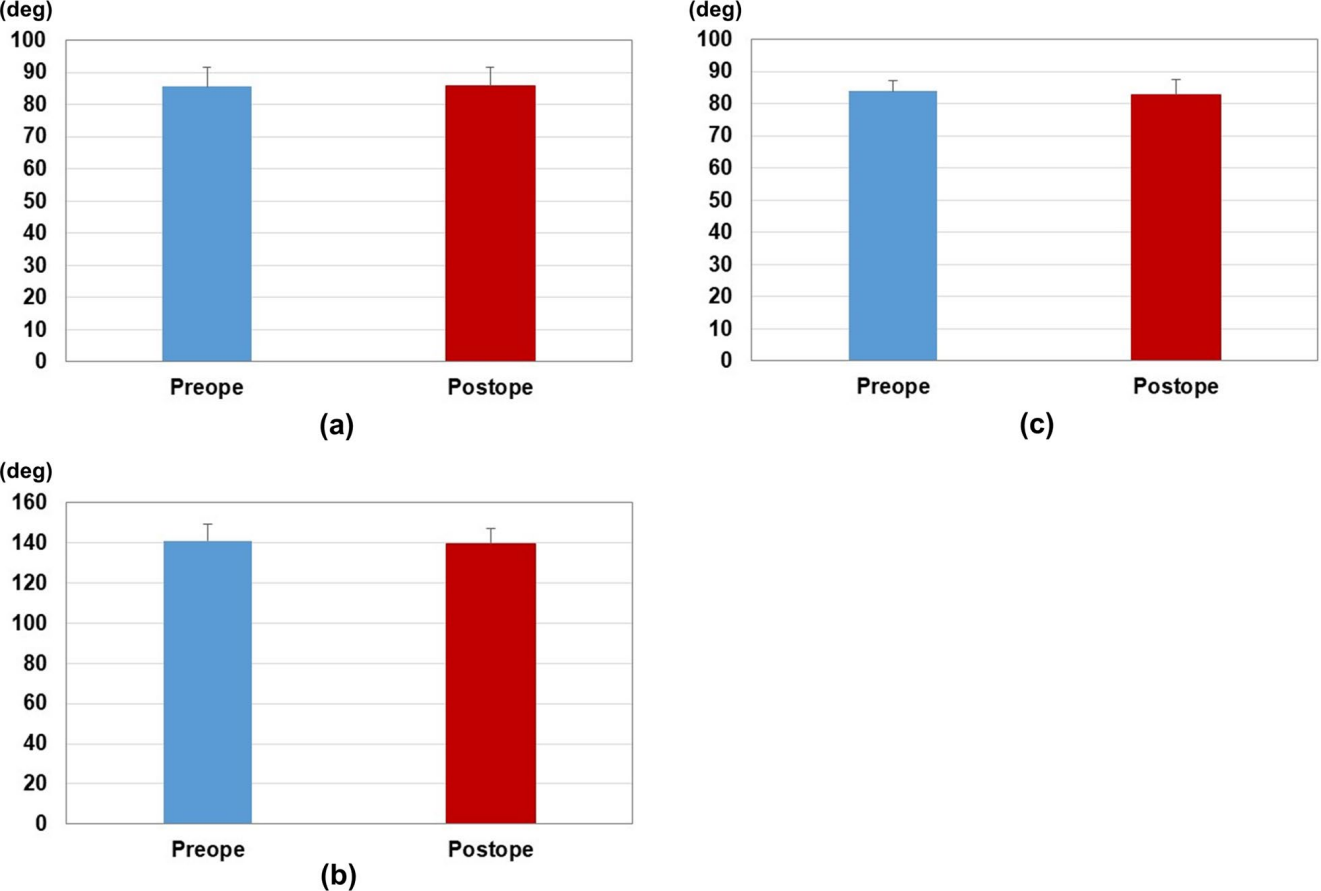
Results of parameter measurements. **a** Results of the coronal angle. **b** Results of the sagittal angle. **c** Results of the axial angle. The blue bar indicates the measurements of the preoperative plan. The red bar indicates the measurements of the postoperative reduction

**Fig. 5:**
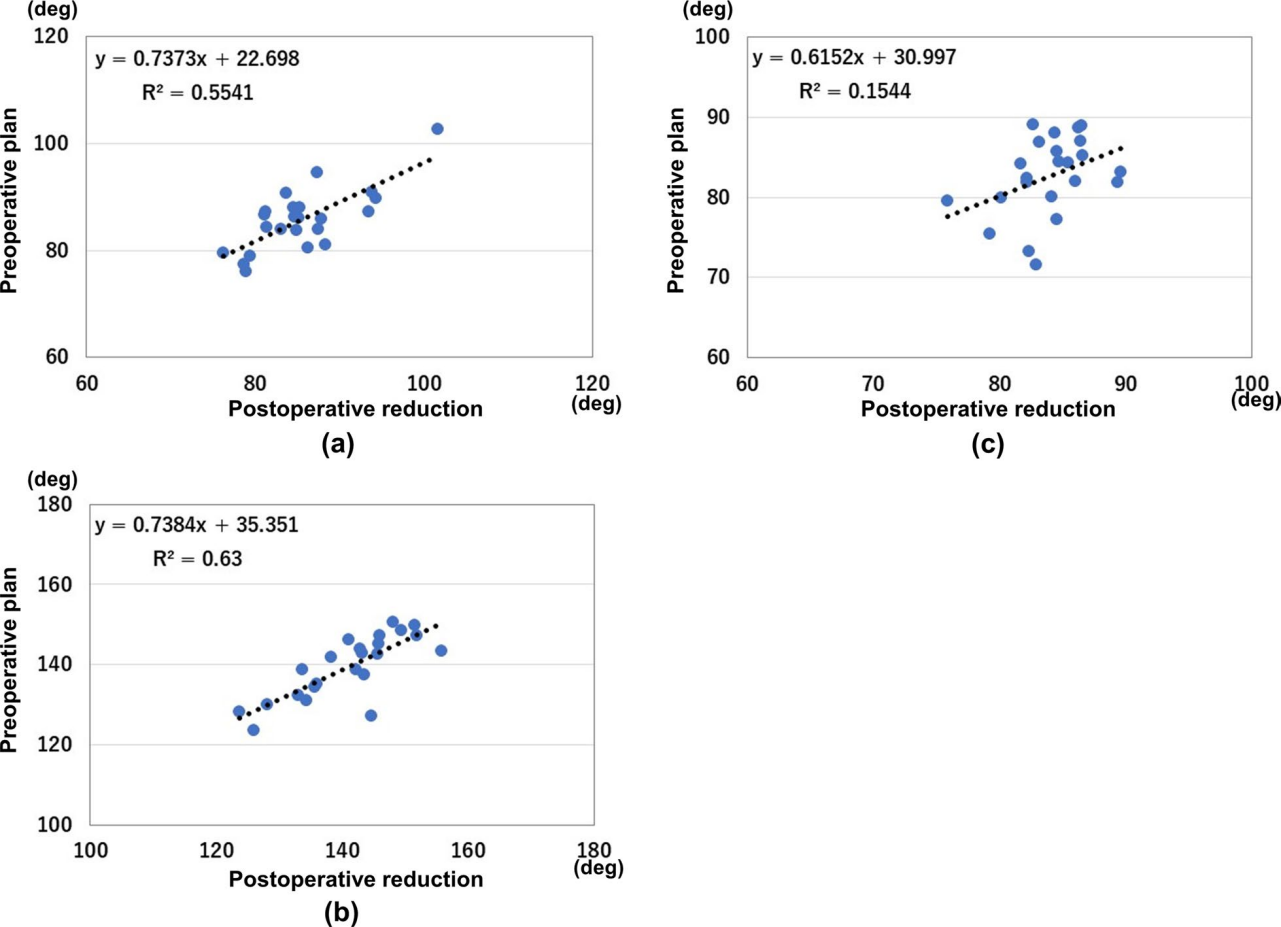
Results of correlations between the preoperative plan and postoperative reduction. **a** Results of the coronal angle. **b** Results of the sagittal angle. **c** Results of the axial angle

The results of measurements are shown in Fig. 4. The results of correlations for angle parameters are shown in Fig. 5. Preoperative planning and postoperative measurement values were CA: 85.6 ± 5.9°/85.8 ± 5.9°, SA: 140.9 ± 8.5°/139.4 ± 7.9°, and AA: 84.0 ± 3.1°/82.6 ± 4.9°, respectively. There were no significant differences between preoperative planning and postoperative measurements. ICCs were CA: 0.75 (*P* < 0.01), SA: 0.78 (*P* < 0.01), and AA: 0.34 (*P* < 0.05). There were good correlations in CA and SA, respectively. There was a poor correlation in AA. Interrater reliabilities were excellent for CA and SA with ICC values of 0.98 and 0.96, respectively. Interrater reliability was good for AA with ICC value of 0.89.

## Discussion

In the surgical treatment of distal humeral fractures, stable anatomic joint reconstruction with osteosynthesis is necessary for proper bone healing and early functional recovery [11]. Since biomechanical studies have demonstrated the benefits of double plating over single plating for proximal and intra-articular fractures of the distal humerus [13–15], the placement of double plates in distal humeral fractures has been recommended in some cases. Conventional preoperative planning with X-ray images has generally been performed by transferring image data onto tracing paper. This was the standard method used by most orthopedic surgeons in clinical practice. In conventional planning, rotational reductions were difficult to assess prior to surgery. In addition, there was no method to three-dimensionally evaluate the interference of distal screws from the inside and outside plates. Advances in image processing technology have led to the development of preoperative planning systems based on the digital processing of image data [15, 18, 20, 22, 23]. The preoperative planning system was previously shown to be useful for visualizing the three-dimensional structure of fractures, judging the feasibility of reduction, and assessing the accuracy of implant selections. In addition, a virtual simulation increased the confidence of trainees and improved their decision making [24].

We previously developed a 3D surgical simulation system for distal humerus fractures. This simulation system is useful for evaluating the reduction shapes of rotational and angular deformations in distal humerus fractures. It also allows for the preoperative selection of appropriate screw directions and lengths. However, there is currently no standard protocol to evaluate the accuracy of the reduction for the 3D preoperative planning. Therefore, we herein attempted to develop a method for evaluating the reduction accuracy in 3D digital preoperative planning for the osteosynthesis of distal humerus fractures. In the original method, the accuracy was assessed from separate images of the preoperative plan and postoperative reduction. Difficulties are sometimes associated with evaluating the accuracy for the correction of rotation using this method. Using a registration algorithm, injured, preoperative planning, and postoperative reduction images were evaluated in the same coordinate system. We propose this method to evaluate the three-dimensional reduction accuracy for the preoperative planning of distal humerus fractures.

The results obtained revealed good correlations for coronal and sagittal parameters and moderate correlation for the axial parameter. This suggests further room for improvements in rotational reduction. In this case series, the majority of incisions were placed posteriorly and the most common approaches were para-tricipital exposure or olecranon osteotomy. In this posterior approach, the posterior surface of the distal humerus bone may be clearly visualized in the surgical field. Therefore, it is relatively easy to evaluate the reduction shape viewed from the coronal direction. In addition, the reduction shape in the sagittal plane may be confirmed by fluoroscopy. On the other hand, difficulties were associated with evaluating the correction of rotation both in the surgical field and fluoroscopy. This may be one reason for the poor correlation of the axial angle. This may need to be improved by creating a reference point on the forearm for a surgical site assessment in future studies. This method and the parameters examined may be useful for confirming the three-dimensional reduction accuracy of preoperative planning in the osteosynthesis of distal humerus fractures.

There are several limitations that need to be addressed. CT scans were needed for 3D preoperative planning. CT has clear advantages in terms of excellent bone and soft tissue contrast and no geometric distortion. However, it exposes the patient to radiation. Precautions need to be taken to reduce radiation exposure, such as scanning elbows away from the trunk. Furthermore, we did not compare the reduction shape with the unaffected side of the patient’s elbow. For assessment of normal anatomical reduction, it may be better to compare the reduction shape with the unaffected side of the elbow. In addition, we did not compare clinical outcomes with the accuracy of reduction in cases without 3D preoperative planning because assessments of reduction based on 3D reference points were only possible when performing 3D preoperative planning. To demonstrate the clinical significance of 3D preoperative planning, the clinical outcomes in different preoperative planning methods need to be examined.

In conclusion, 3D preoperative planning for distal humeral fractures showed the good correlations of coronal and sagittal angles, but the relatively poor correlation of axial angle. This may be attributed to an inability to assess the rotation angle during surgery. We propose the measurement indices shown in the present study as three-dimensional evaluation parameters for the reduction of distal humerus fractures.

## Data Availability

The datasets used and/or analyzed during the present study are available from the corresponding author upon reasonable request.

## Abbreviations

3D: Three-dimensional
CT: Computed tomography
AO: Arbeitsgemeinschaft für Osteosynthesefragen
ICC: Intra-class correlation coefficients
